# First Step into Praziquantel Raw Material Color Change Investigation: The Role of Thermal, Spectroscopic, and Microscopic Techniques

**DOI:** 10.1021/acsomega.5c05829

**Published:** 2025-10-06

**Authors:** Livia Deris Prado, Silvia Lucia Cuffini, Pedro Pôssa de Castro, Francisco Alexandrino-Júnior, Lara Melo Campos, Giovanni Wilson Amarante, Luiz Fernando Cappa de Oliveira, Helvécio Vinícius Antunes Rocha

**Affiliations:** † Laboratory of Micro and Nanotechnology, Center of Technological Development in Health, 340747 Oswaldo Cruz Foundation, Rio de Janeiro 21040-900, Brazil; ‡ Laboratory of Analytical Development and Validation, Farmanguinhos Oswaldo Cruz Foundation, Rio de Janeiro 21040-900, Brazil; § Post-Graduate Program in Engineering and Science of Materials, Federal University of São Paulo, São José dos Campos, São Paulo 12231-280, Brazil; ∥ Research Group on Synthetic Methodologies, Department of Chemistry, Federal University of Juiz de Fora, Juiz de Fora 36036-900, Brazil; ⊥ Chemistry Department, 28113 Federal University of Juiz de Fora, Juiz de Fora 36036-900, Brazil

## Abstract

Praziquantel (PZQ) is an anthelmintic agent used worldwide for the treatment of schistosomiasis. PZQ is used as a racemate, and it is practically insoluble in water. The PZQ racemate is a white to nearly white crystalline powder, and few studies showed a color change from white to pink. However, no special attention has been given to this matter. The present study aimed at a comprehensive understanding of PZQ change in color because a significant impact on its dissolution was observed. We discuss a series of analytical techniques, and we emphasize the importance of understanding solid state properties together with the conventional quality control evaluation. Two batches of PZQ raw material with different colors (white and light pink) were used. The dissolution profiles of the samples and the wettability were significantly different, and in addition, the pink color sample, when it came into contact with an acid medium, turned white. Because of these results, the presence of another phase in the pink sample was investigated and confirmed by powder X-ray diffraction. We were able to isolate the unknown phase for the first time, and with the characterization using Raman, infrared, and nuclear magnetic resonance, we proved that the pink color was related to an impurity with low crystallinity. It was observed through microscopy that this impurity with low crystallinity after the contact with the acid medium crystallizes, causing the pink color to turn white. Also, we showed that depending on the HPLC method, this impurity cannot be detected, which is critical for quality control.

## Graphical abstract

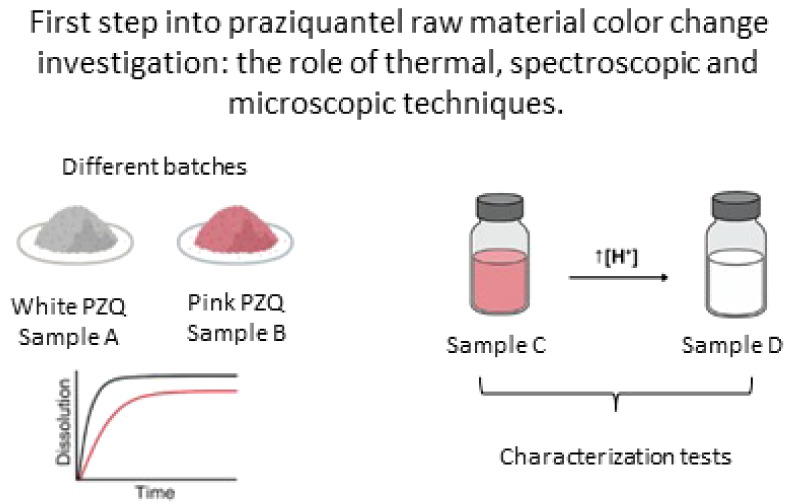

## 1 Introduction

Praziquantel (PZQ), an anthelmintic agent, is recognized as the drug of choice for the control and treatment of schistosomiasis.^1^ It is used worldwide, especially in developing countries, and due to its efficacy and safety, it is included in the World Health Organization’s model list of essential drugs.^2^

Praziquantel’s molecular structure presents an asymmetric center at the position marked with an asterisk in Figure 1. In therapy, PZQ is used as a racemate. The l-(−)-enantiomer is the eutomer and has the (R) configuration.^3^ Administration of the pure eutomer resulted in fewer side effects than the racemate.^4^ The inactive (+)-enantiomer is associated with side effects and is also primarily responsible for the extremely bitter taste of the tablet.^3–5^

**Figure 1:**
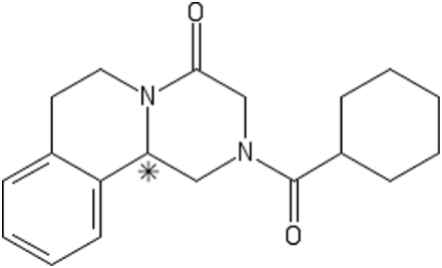
Molecular structure of praziquantel.

A PZQ racemate is a lipophilic drug practically insoluble in water^3^ classified as a Class II drug^6^ according to the Biopharmaceutics Classification System (BCS).^7^ PZQ low solubility of PZQ in aqueous solution results in the necessity for large doses to achieve adequate serum concentrations. This factor (large tablet size) combined with bitter taste contributes to the problem of adherence to treatment with PZQ in the affected communities.^8,9^

The PZQ racemate is a white to nearly white crystalline powder, with melting at 136–142 °C (USP, 2019). There are few studies showing the color change of PZQ from white to pink.^10–12^ The physical appearance is a symbol of both the pharmaceutical quality of the product and the good manufacturing practices of its manufacturer. A visual aspect which meets requirements is thus mandatory and officially cited as a critical quality attribute.^13^

A previous study reported implants containing PZQ with different drug loadings with a slight pink. The authors speculated that the reason for this discoloration was the decomposition of PZQ during the fabrication process, which involved heat, shear, and pressure. To verify the hypothesis, PZQ extracted from the implants and the PZQ raw material were evaluated by nuclear magnetic resonance (NMR) and high-performance liquid chromatography (HPLC). Both samples had identical NMR spectra and the same retention time. No additional peak was observed in the HPLC. For the authors, these results demonstrated that PZQ was stable during the fabrication process, and the change in color was not further studied.^10^

Discoloration of some PZQ tablets was also reported in Sudan, while being retained under the appropriate storage conditions and during the provisional shelf life of the product. With this, and due to environmental conditions in Sudan, some authors investigated the photo and thermal stability of PZQ. PZQ was found to be highly stable toward thermal decomposition in its solid form and photolabile when exposed to ultraviolet radiation or naturally, when directly exposed to sun rays for a long period. Praziquantel proved to be a photo- and thermo-labile drug when studied in aqueous media.^14^

A document published by the Food and Drug Administration (FDA) describes batches of PZQ injections released in 2006 from Teva Animal Health with an unapproved specification. They failed the stability specification (clear, colorless to tinted) because they were pink. Subsequent batches of the product, released in 2006 and 2007, had appearance results consistent with the confirmed failures, and quality control failed to investigate the results. This change in color has never been evaluated by the firm for its potential impact on efficacy.^15^

Even though PZQ is widely approved for the treatment of schistosomiasis, there is little information concerning the pink color appearing in the raw materials and its products. Thus, the present study aims to provide a comprehensive understanding of PZQ color change from the observation of significant alterations in dissolution profiles and wettability of different white and pink samples of PZQ. Due to this possible impact on drug quality, we discuss a number of analytical techniques needed to understand the characteristics of PZQ and to detect, during quality control testing, the new phase observed. Given the potential implications for drug quality and efficacy, we discuss and explore a range of analytical techniques suitable for understanding the characteristics of praziquantel and identifying the newly observed phases during quality control assessments.

## 2 Materials and Methods

Two batches of PZQ raw material, previously approved for pharmacopoeial criteria (in quality control) and kept in a warehouse, were used for the study. These raw materials were initially selected for evaluation in an internal dissolution test study project. However, it was found that the samples had different colors: white (sample A, already micronized) and light pink (sample B, already micronized).

After the dissolution study, in which solubility and wettability were previously evaluated, the samples were studied by HPLC. Following these tests, characteristics that could be related to color change and dissolution were investigated.

### 2.1 Initial Study for Dissolution Evaluation

#### 2.1.1 Solubility Tests

Solubility tests were performed to select the appropriate dissolution media. Suspensions were prepared with samples A and B in 10 mL of water, HCl 0.1 N pH 1.2, acetate buffer pH 4.5, phosphate buffer pH 6.8, and HCl 0.1 N pH 1.2 with SLS 0.1% and 0.3% (w/v). Media with different pH were prepared.^16^ The samples were kept under agitation for 48 h, after which the solids were filtered, and the supernatants were evaluated by ultraviolet–visible (UV–vis) spectrophotometry at a wavelength of 210 nm using a UV-1800 spectrophotometer (Shimadzu, Japan). The saturation concentrations were obtained from analytical curves previously evaluated in each medium.

#### 2.1.2 Wettability Tests

Wettability tests were conducted for samples A and B. The test was performed by means of the contact angle by the sessile drop method with a DSA 100 (Krüss, Germany). The pellets were prepared with the aid of an ICL press, model 1 Ton EZ, flat 10 mm in diameter weighing approximately 300 mg under a pressure of 800 psi for 1 min. For the determination of the contact angle, approximately 8 μL of liquid (saturated media with praziquantel) was used at room temperature, with an application rate on the tablet surface of 100 μL·min^–1^. Water, HCl 0.1 N, HCl 0.1 N with SLS 0.1% and 0.3% (w/v) were used as media. The analyses were performed in triplicate. The contact angle was measured at time zero, immediately after drop deposition, and its time evolution was not monitored.

#### 2.1.3 Dissolution Tests

Powder and intrinsic dissolution tests were performed for samples A and B in Evolution 6100 equipment (Distek, USA). The medium used was HCl 0.1 N pH 1.2 with SLS 0.3% at 37 °C. Aliquots of 10 mL were collected at certain time intervals and quantified using a UV-1800 spectrophotometer (Shimadzu, Japan) without dilution at a wavelength of 210 nm. The concentrations were obtained from analytical curves previously constructed. The test was performed in triplicate. For powder dissolution, approximately 600 mg were inserted in the vessels at a stirring rate of 50 rpm (apparatus 2). For the intrinsic dissolution, 100 mg were subjected to 800 psi for 1 min using a press, model 1 Ton EZ (ICL, USA). The selected pressure allowed the formation of a nondisintegrating compact. A compress of 0.5 cm^2^ was used with a stationary disc system at a stirring rate of 100 rpm with flat vessels.

### 2.2 Separation of the Unknown Phase

After the initial study, it was possible to imagine that sample B contained PZQ, as in sample A, and a different phase. For the separation of this unknown phase, sample B was suspended in ethanol and kept under agitation for 10 min. After this period, the solid was filtered and dried at 40 °C; the solid was named sample C. Using sample C, another test was also performed by suspending it in HCl 0.1 N pH 1.2 for 10 min. After this period, the solid was filtered and dried at 40 °C; the solid was named sample D.

### 2.3 Characterization Tests

#### 2.3.1 Powder X-ray Diffraction

Samples A, B, and C were evaluated using a D8 Advance diffractometer (Bruker, Germany) equipped with a LYNXEYE XE detector, using Cu Kα radiation (λ = 1.5418 Å). The voltage and current during the experiment were 40 kV and 40 mA, respectively. A step size of 0.02° and a 0.01 s step time were used. Samples were scanned from 3° to 40°.

#### 2.3.2 High Performance Liquid Chromatography (HPLC)

In the first evaluation using HPLC, 18 mg of the samples were dissolved in 100 mL of a mixture of water–acetonitrile (60%:40%, v/v) (HPLC grade). The solution was filtered with a disposable syringe filter of 0.45 μm regenerated cellulose prior to HPLC analysis. HPLC was performed using an LC-10A (Shimadzu, Japan) with LC-10AD pumps, a DGU-12A degasser, an SIL-10AD automatic injector, a CTO-10A column oven (30 °C), a SPD-M10A photodiode array detector, and a Supelcosil C18 column (200 cm × 4.6 mm, 3.0 μm). Samples were eluted with water–acetonitrile (60%:40%, v/v) (HPLC grade) at 1 mL·min^–1^.

In the second evaluation using HPLC, 1.20 mg of sample was added to 8 mL of a solution containing 2-(*N*-morpholino)­ethanesulfonic acid (MES) buffer pH 5-acetonitrile (25%:75%, v/v). The solution was filtered with a disposable syringe filter of regenerated cellulose 0.45 μm prior to HPLC analysis. The HPLC system (Shimadzu, Japan) consisted of two 20ADXR pumps, a DGU-20A5R degasser, a SIL-30AC automatic injector, a CTO-20A column oven (30 °C), an SPD-M20A photodiode array detector, and an ACE C18-PFP column (150 cm × 3.0 mm–3.0 μm). Upon injection of 10 μL of sample, a gradient consisting of mobile phase A (water) and mobile phase B (acetonitrile) was initiated at a flow rate of 1 mL·min^–1^ using a time program.

#### 2.3.3 Infrared and Raman Spectroscopy

FTIR spectroscopy was performed using a Nicolet 6700 spectrometer (Thermo-Nicolet, USA) attached to an attenuated total reflectance accessory. Spectra were recorded over a range of 4000–650 cm^–1^ with a mean of 32 scans at a resolution of 4 cm^–1^. Raman spectra were recorded in a FT-Raman RFS/100 from Bruker Optics (Germany) using 1064 nm exciting radiation (Nd:YAG laser Coherent Compass 1064-500N) and a Ge detector. Laser power was kept at 300 mW. Spectra were recorded by accumulating 512 scans at a resolution of 2 cm^–1^, over a range between 4000 and 50 cm^–1^. All spectra were obtained at least twice in order to avoid any loss of integrity in the samples.

#### 2.3.4 Thermogravimetric Analysis

Thermogravimetric analyses of samples A–C were obtained using an 851e instrument (Mettler Toledo, Switzerland) operating at 10 °C·min^–1^ heating rate from 30 to 600 °C in a nitrogen flow rate at 50 mL·min^–1^. Approximately 10 mg of the sample was heated in an alumina crucible. The experiments were performed in duplicate for each sample.

#### 2.3.5 Scanning Electron Microscopy

Scanning electron microscopy (SEM) was used to observe changes in the pressed samples before and after intrinsic dissolution. Also, after the separation of the unknown phase, SEM was used to compare the particle morphology of the three samples. Samples were spread on a sample holder and then coated with gold by a SCD 050 Sputter coater (Bal-Tec, Liechtenstein) for evaluation on the microscope; Quanta 400 (FEI, USA) and TM3030Plus (Hitachi, Japan) equipment were used.

#### 2.3.6 Nuclear Magnetic Resonance (NMR)

The NMR spectra of samples A–D were recorded at 298 K in an Avance III spectrometer (Bruker, Germany). The ^1^H NMR spectra were recorded at 500 MHz, and ^13^C NMR spectra were recorded at 125 MHz. The samples (15 mg) employed in the analysis were previously dissolved in 0.5 mL of deuterated trifluoroacetic acid (TFA-*d*). The chemical shifts are reported in parts per million relative to the solvent residual peaks. Monodimensional ^1^H spectra were measured with 64 scans and a recycle delay of 5 s. ^13^C NMR spectra were obtained with proton decoupling during acquisition time, a recycle delay of 3 s, and 6000 scans. DEPT135 and two-dimensional NMR methods (^1^H–^1^H COSY, ^1^H–^13^C HSQC, and ^1^H–^13^C HMBC) were also employed and are available in the Supporting Information.

## 3 Results and Discussion

### 3.1 Initial Dissolution Studies

The two PZQ samples, before the expiration date and previously approved internally (by the Quality Control Department), were studied by solubility and dissolution tests to verify the suitability of the dissolution method and the influence of the raw material characteristics.

#### 3.1.1 Solubility and Wettability Studies

Solubility of samples A and B in various media is presented in Table 1. The results obtained from solubility studies performed with the PZQ pure drug are related to its physicochemical properties. Since PZQ is a very lipophilic drug, its solubility was poor in all media (without the presence of surfactant).

**Table 1:** Solubility (μg·mL^–1^) and Contact Angle (°) Results of PZQ Samples A and B in Different media^a^ ^b^

	Sample A		Sample B	
Media	Solubility	Contact angle	Solubility	Contact angle
Water	264.8 ± 13.5	75.2 ± 0.6	253.9 ± 7.3	94.6 ± 2.2
Acetate buffer pH 4.5	195.12 ± 12.93	-	203.31 ± 13.43	-
Phosphate buffer pH 6.8	201.57 ± 18.87	-	231.25 ± 11.11	-
HCl 0.1 N pH 1.2	268.54 ± 4.01	77.9 ± 3.7	233.80 ± 10.61	87.2 ± 0.7
HCl 0.1 N pH 1.2 + LSS 0.1% (w/v)	595.76 ± 27.61	17.4 ± 0.7	546.18 ± 54.80	66.8 ± 1.6
HCl 0.1 N pH 1.2 + LSS 0.3% (w/v)	1349.09 ± 44.30	*	1285.71 ± 48.43	59.8 ± 4.3

a –Not performed.

b Not possible to measure.

Among the common media described in USP (2019)^16^ to evaluate dissolution at different pH, both samples presented higher solubility in HCl 0.1 N pH 1.2, and the solubility value of PZQ raw material is in accordance with the literature.^17^ The medium with HCl 0.1 N was chosen, and sodium lauryl sulfate (SLS) was added to improve solubility. As expected, the solubility of both samples increased with the concentration of SLS.

The contact angle measurement results are shown in Figure 2. Clearly, the contact angle was higher for sample B in all media. Also, the results are in good agreement with the solubility and indicate an increase in the wettability (lower contact angle) for sample A. Because the solubility was higher in HCl 0.1 N pH 1.2 with LSS 0.3% (w/v), this medium was chosen for dissolution tests.

**Figure 2:**
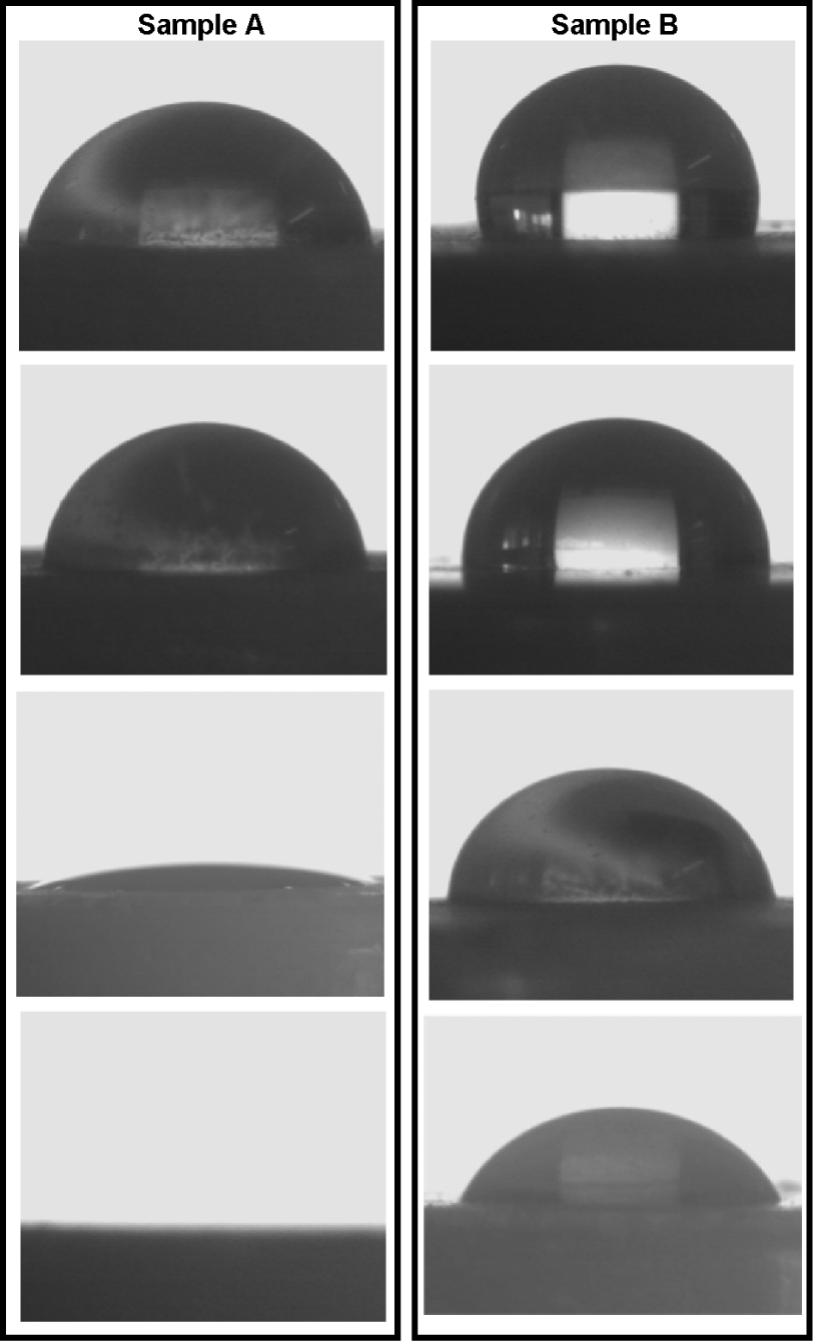
Contact angle measurement results of samples A and B in different saturated media. From top to bottom: water, HCl 0.1 N pH 1.2, and HCl 0.1 N pH 1.2 with SLS 0.1% (w/v) and with SLS 0.3% (w/v).

#### 3.1.2 Powder and Intrinsic Dissolution Tests

The powder dissolution profiles of samples A and B are depicted in Figure 3A. A thorough analysis of these data reveals a similar pattern for both materials, i.e., an immediate dissolution upon contact with the dissolution medium, followed by a first-order kinetic process that eventually reaches a plateau. Despite the similarity, the rate and extension of the dissolution process differ remarkably between these samples. Therefore, to obtain a more comprehensive understanding of this behavior, different mathematical models were fitted to dissolution data. Based on statistical parameters and visual analysis, a first-order model^18^ with *F*_max_ and *F*_0_ was identified as the most suitable model (Table 2and Figure 3A). Its parameters were determined using nonlinear regression with the least-squares method. The fitting was performed in Microsoft Excel using the Solver tool configured to minimize the sum of squared errors (SSEs) with the GRG Nonlinear algorithm. As a result, the values of the parameters *F*_0_, *F*_max_, and *k* were optimized to achieve the best fit, yielding eqs 1 and 2, which accurately describe the dissolution profiles of samples A and B (Figure 3A).

1 $$F=57+32\left(1-e^{-0.13.t}\right)$$

2 $$F=7+71\left(1-e^{-0.05.t}\right)$$

**Table 2:** A Summary of the Mathematical Models Used for Fitting Dissolution Data, Including Their Respective Equations and Key Statistical Parameters

Model	Equation	Sample	*R* ^2^	*R* ^2^ Ajus	SSE^a^
1st order	*F* = 100 (1 – *e* ^*–k.t*^)	A	–2.43	–3.00	875.05
		B	0.67	0.62	1017.46
1st order with *F*_max_	*F* = *F*_max_ (1 *– e^–^* ^*k*.*t*^)	A	0.86	0.80	39.34
		B	0.98	0.97	14.39
1st order with Fmax & F_0_	*F* = *F*_0_ + *F*_max_ (1 – *e* ^*–k.t*^)	A	0.99	0.98	3.24
		B	1.00	1.00	1.62

a Sum of squared error. In all models, *F* is the fraction (%) of PZQ dissolved in time *t*, *F*_0_ is the initial fraction of PZQ in the dissolution medium resulting from a burst release, *F*_max_ is the maximum fraction of the drug released at infinite time, and *k* is the first-order dissolution constant.

**Figure 3:**
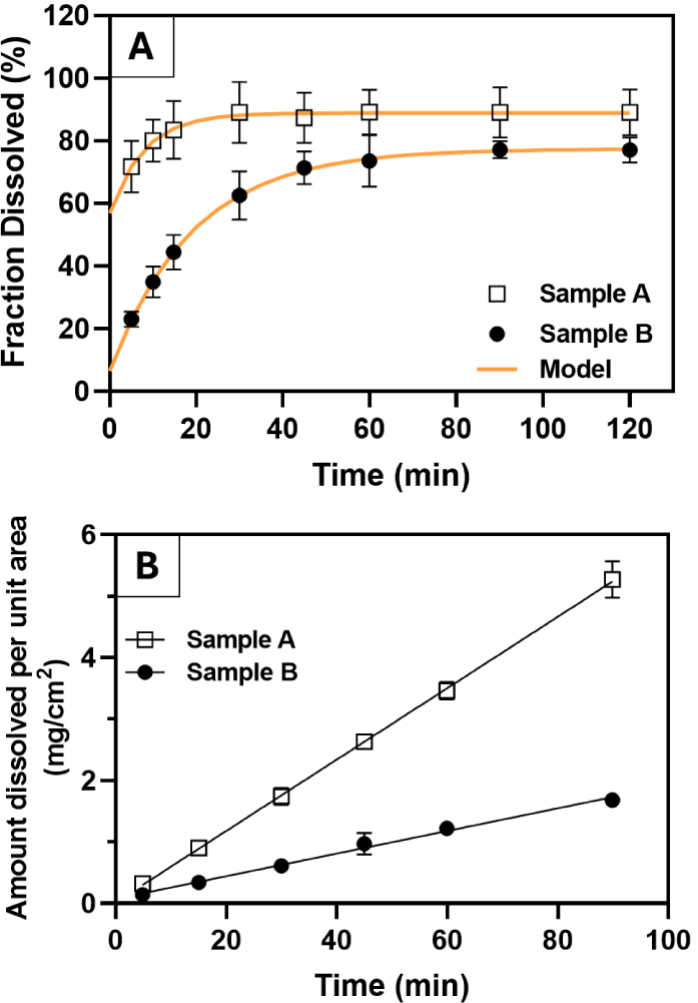
Powder dissolution (top) and intrinsic dissolution (bottom) of samples A (□) and B (●).

In light of the mathematical model and the experimental data, it could be inferred that sample A exhibited rapid dissolution, with a burst release corresponding to 57% of the dose and 85% dissolution in less than 30 min, whereas sample B did not exhibit a significant burst release (<10%) and dissolved less than 80% of the dose throughout the test. In fact, the dissolution efficiency^19^ of samples A and B, calculated from the area under the curve of each plot (Figure 3A), was 85.7 ± 7.8% and 67.0 ± 3.5%, respectively.

Given the color differences observed between the PZQ samples, the possibility of different crystal structures was initially considered. Therefore, to characterize the crystal forms regarding their dissolution profiles and to eliminate the influence of the particle size and agglomeration, intrinsic dissolution tests were carried out (Figure 3B). The results revealed a strong linear correlation between time and concentration (*R* ^2^ > 0.99). The intrinsic dissolution rates (IDR), calculated from the slope of each plot (Figure 3B), were determined to be 44.9 ± 1.0 and 17.7 ± 0.3 μg·cm^–2^·min^–1^ for samples A and B, respectively.

A 2.5-fold increase in IDR values was observed for sample A, which was corroborated by the mathematical modeling of dissolution test, as a dissolution rate (*k*) 2.6-fold higher than sample B was obtained for sample A (eqs 1 1 and 2). Additionally, upon removal of the tablet from the intrinsic dissolution accessory for posttest cleaning, the tablet, initially pink, exhibited the area that had been in contact with the dissolution medium as white-colored (Figure 4).

**Figure 4:**
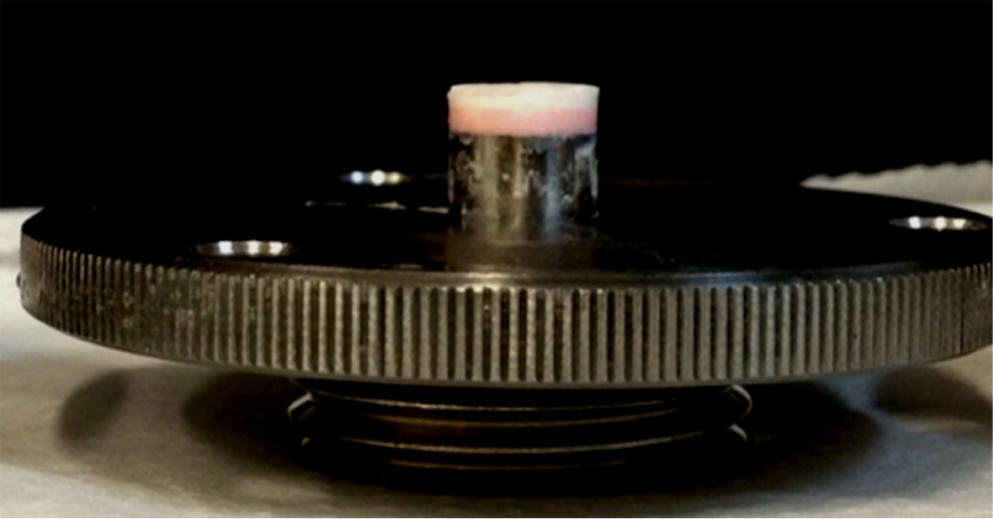
Photograph of sample B after intrinsic dissolution, showing the change from light pink to white in the region of the tablet that came into contact with the acid dissolution medium. Photograph courtesy of Livia Prado. Copyright 2023.

Thus, the results from the initial project evaluating PZQ dissolution led to two questions being posed: whether the observed differences between samples A and B, aside from color, were due to another solid form of PZQ or to a possible degradation product; and (2) what occurred with sample B, which changed from pink to white upon contact with the dissolution medium.

### 3.2 Characterization Tests

#### 3.2.1 Powder X-ray Diffraction

Powder X-ray diffraction (PXRD) was selected as a technique to evaluate the possible presence of a different crystal structure of PZQ in sample B. PZQ presents solved crystalline structures, reported in the literature: the racemate, a hydrate, and some cocrystals.^20^ With these determined structures, it was possible to compare the position of the diffraction peaks with the samples (Figure 5). Sample A presents characteristic peaks of the (RS)-PZQ structure reported in the literature.^20^

**Figure 5:**
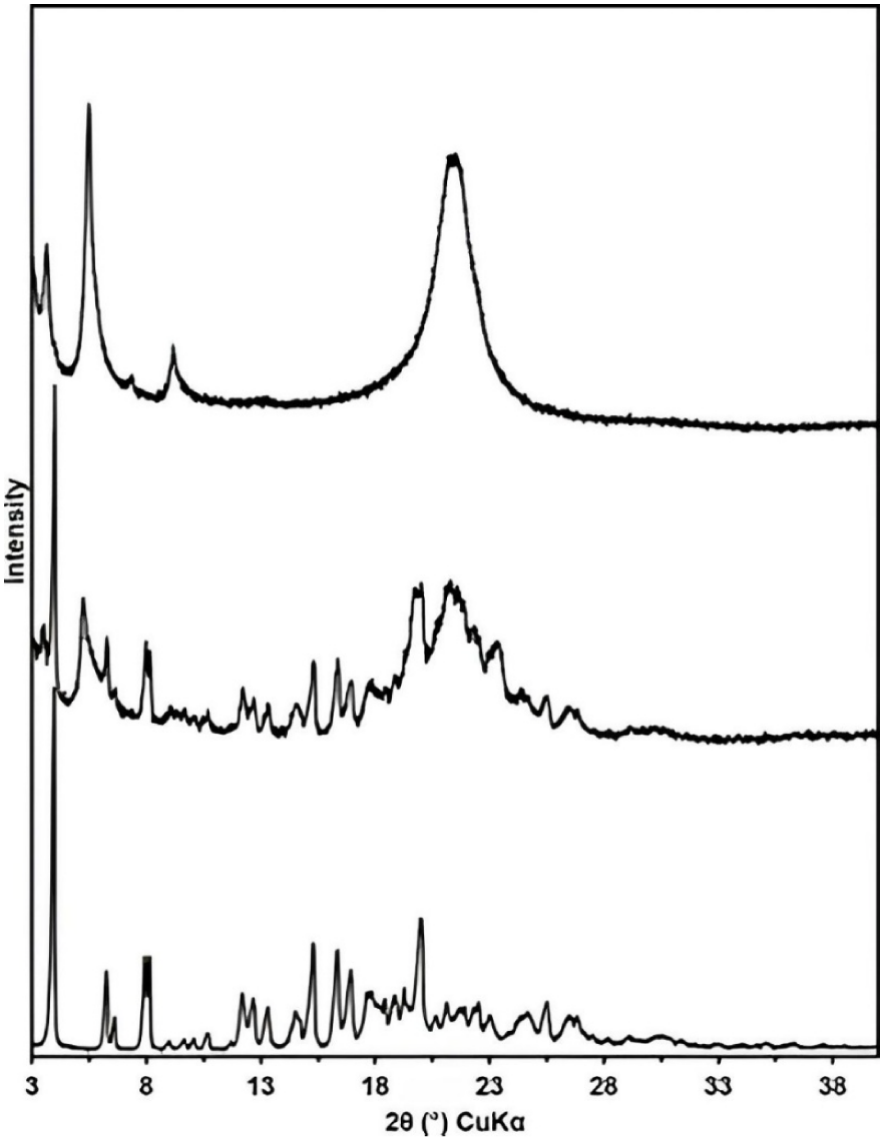
X-ray powder diffraction patterns of the PZQ samples. From bottom to top: samples A, B, and C.

In relation to sample B, there was an enlargement in the diffraction peaks and the appearance of a new peak, which did not exist in sample A, in 5° in 2θ. This peak was investigated, and it was not compatible with the other reported PZQ structures.^20,21^

The separated phase, sample C, was also evaluated. One can see a different PXRD pattern compared to sample A. However, it seems that sample C was still contaminated with pure PZQ (present in sample A). With sample C, we could describe the peak in 5 ° in θ, there are also fewer diffraction peaks, and these were very broad. This is related to the low crystallinity of the sample. In microstructural terms, the progressive broadening of peaks from A → B → C indicates a reduction in the crystalline coherence length and a possible increase in microstrains.

It is also important to note that a large part of the PZQ papers,^22,23^ including the previously cited one about the implants in which pink coloration was observed,^10^ shows the PZQ XRD patterns from 5° onward. This results in a loss of information because, as can be seen in Figure 5, the peak appearance in samples B and C occurs at this angle.

Although many papers are being published with the aim of the development of new crystalline or amorphous structures of PZQ^24,25^ when PXRD is a powerful characterization technique, the main objective of its use in the present research was to evaluate if the color changing had some correlation with structural transition or disorganization. Moreover, it is a complementary technique that, in combination with DSC, TGA, and FTIR, can present a better understanding of the process observed.

#### 3.2.2 Evaluation by HPLC

The results of the first test performed by HPLC are shown in Figure 6. Samples A and B were evaluated using the first method, with the samples being dissolved in a mixture of water and acetonitrile (United States Pharmacopoeia 42 indication). With this method, we observed the same retention times: a sharp peak at 6.8 min and a small peak at 4.2 min. The only difference between the two chromatograms was the intensity of absorbance, which was lower for sample B.

**Figure 6:**
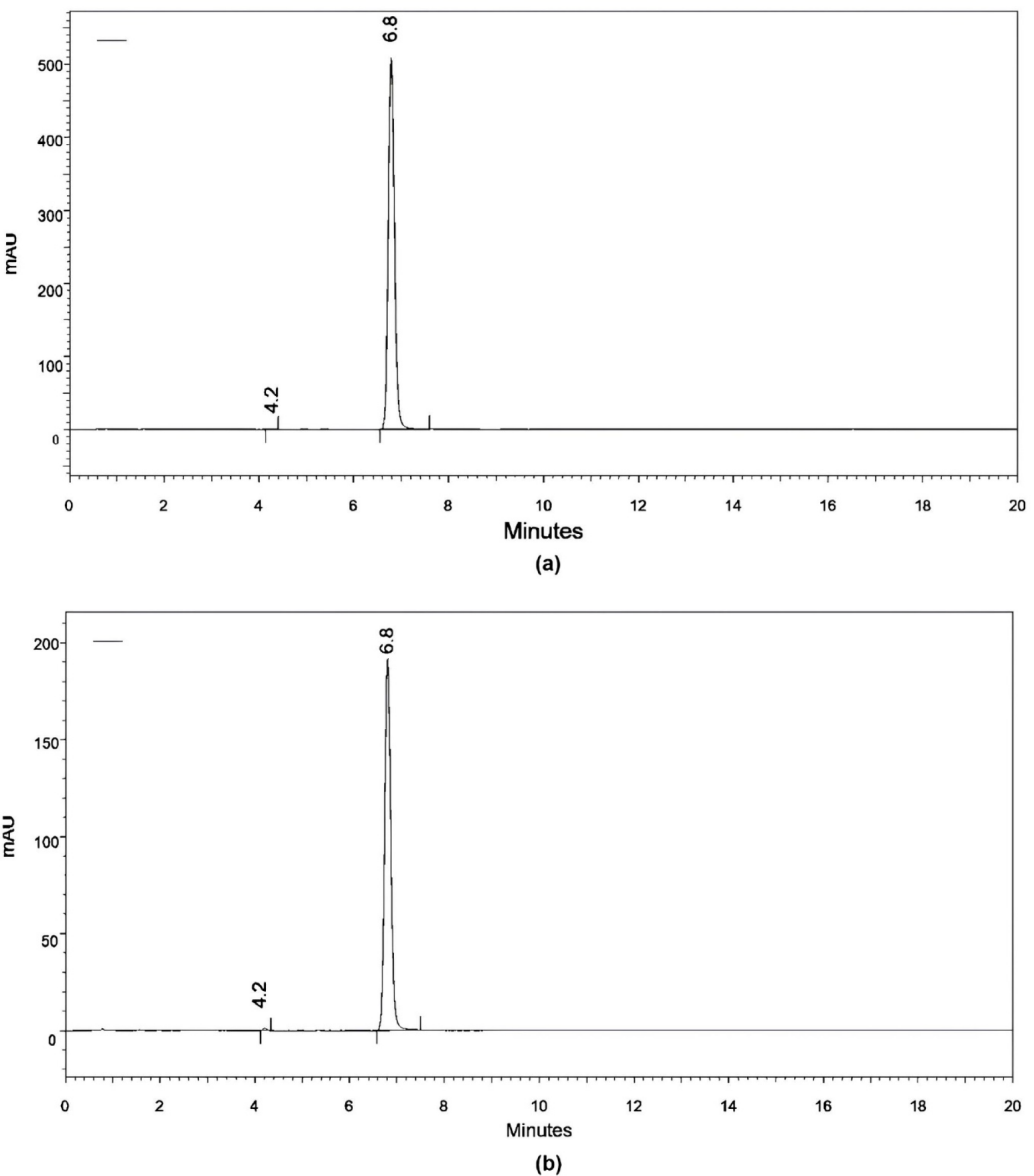
First HPLC chromatograms of samples A and B at 210 nm.

Investigating this fact, the possibility was raised that sample B, especially the unknown phase, was not completely dissolved before the injection. Therefore, a second HPLC test was conducted using a mixture of MES buffer and acetonitrile. This buffer was the only medium that completely dissolved sample B.

The results of the second test (Figure 7) showed differences in the chromatograms, and with these methods, it was possible to also evaluate sample C. A first peak (peak 1) appeared in all samples, due to (RS)-PZQ. In sample A, we observed the presence of very small peaks (2 and 3) due to the presence of impurities that are also present in samples B and C. It is interesting to note that in samples B and C, there was a peak that was not present in sample A, and there was also a reduction in the intensity of absorbance, indicating a lower content of PZQ.

**Figure 7:**
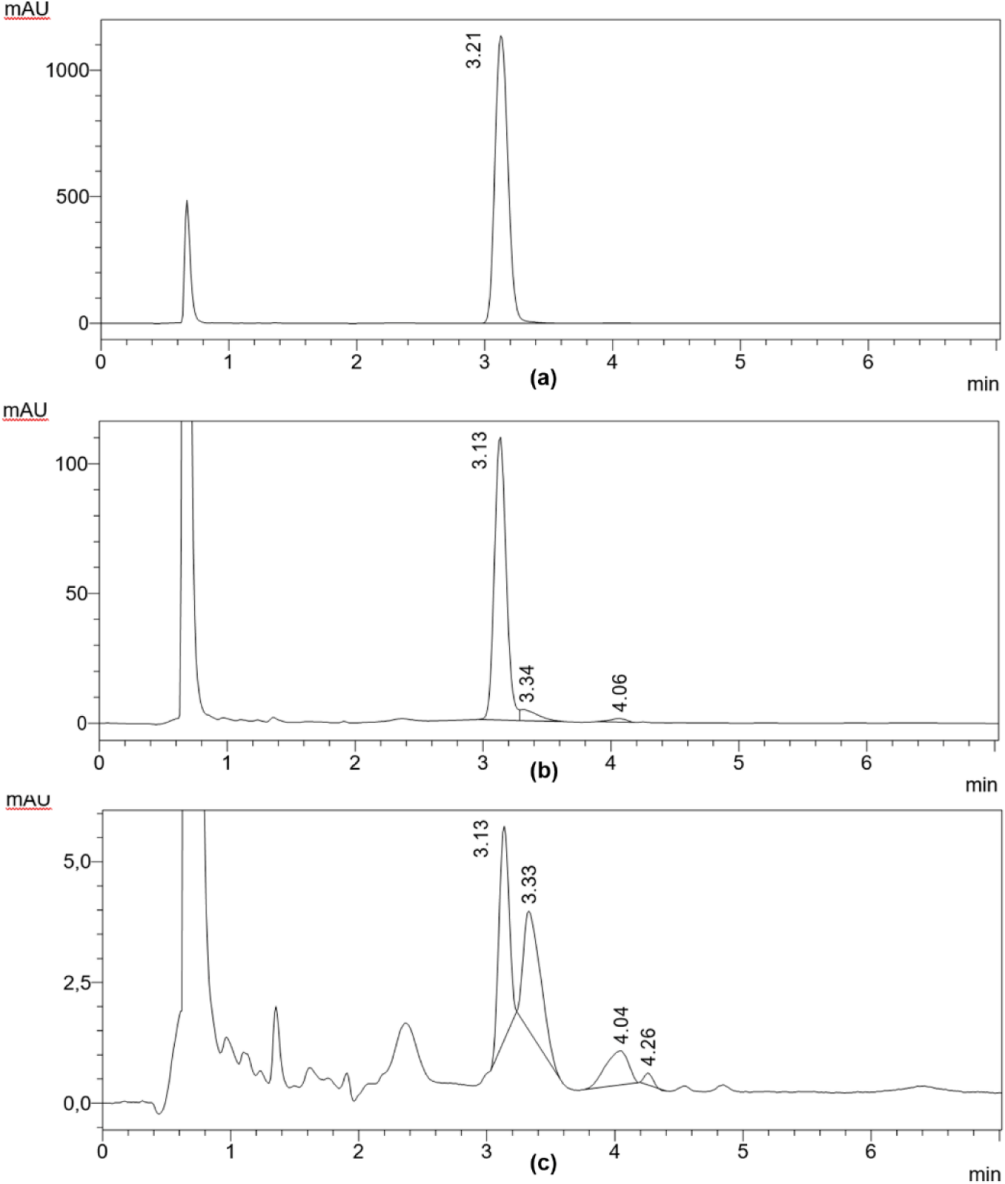
Second HPLC chromatograms of samples A–C at 210 nm.

#### 3.2.3 IR and Raman

It is important to first discuss the spectra of the obtained samples and compare them with the literature data. A comprehensive investigation on the solid-state spectroscopic properties of both conformers of praziquantel, including molecular modeling, was recently reported.^26^ The most relevant vibrational marker for both conformers is the ν­(C=O) stretching mode, observed in the 1630–1660 cm^–1^ region. Since praziquantel contains two distinct carbonyl groups, one attached to the cyclohexyl moiety and the other to the heterocyclic ring-two characteristic carbonyl bands are expected. According to the vibrational assignment by Borrego-Sánchez and coworkers, these bands appear at 1638 and 1671 cm^–1^ for the syn conformer and at 1636 and 1663 cm^–1^ for the anti conformer. In our experimental spectra (Figure 8a–c), the ν­(C=O) bands are observed at 1625 and 1647 cm^–1^, slightly lower than the calculated values, likely due to intermolecular interactions in the crystal structure. In sample C, the carbonyl bands are absent, which, together with HPLC results, confirms the degradation of praziquantel.

**Figure 8:**
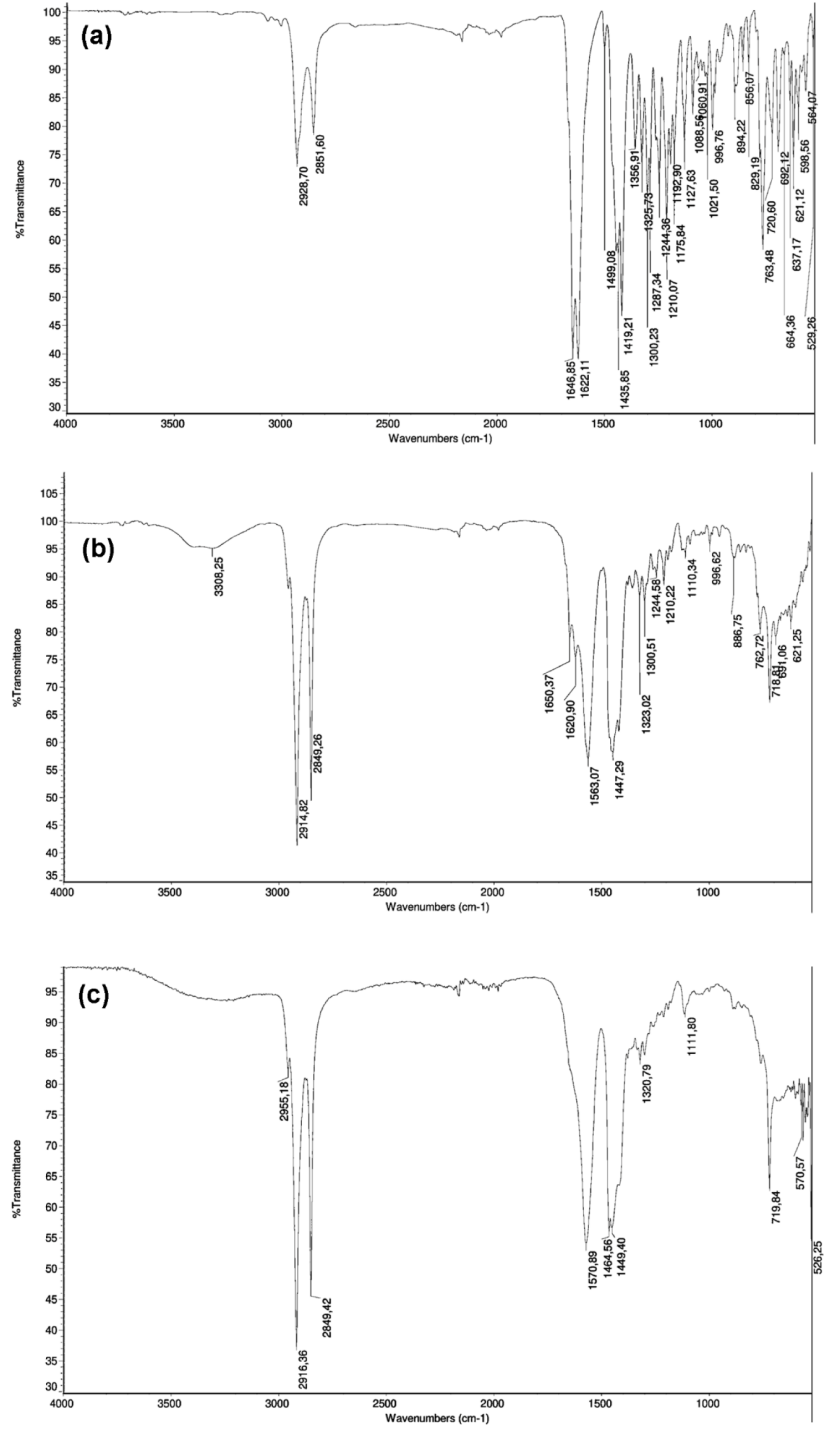
FT-IR spectra of the PZQ samples. From bottom to top: A, B, and C.

Another relevant feature is the C–H stretching region (3000–2700 cm^–1^), where changes in intensity, position, and number of bands suggest alterations in the molecular structure. Differences in band intensities can be correlated with the two types of C–H bonds: those closer to the aromatic ring appear at higher frequencies, whereas those near the cyclohexyl moiety appear at lower frequencies, consistent with literature reports.^26^

Additional bands are observed in the 1100–1400 cm^–1^ region, where δ­(CH), γ­(CH), and ν­(CN) modes may overlap. However, none of these bands provide as definitive a marker as the carbonyl groups, which unambiguously reflect the structural changes occurring in the samples. Overall, the combination of IR and Raman spectra (Figure 9) allows a clear assessment of the solid-state structure of praziquantel in the obtained samples and highlights the degradation in sample C.

**Figure 9:**
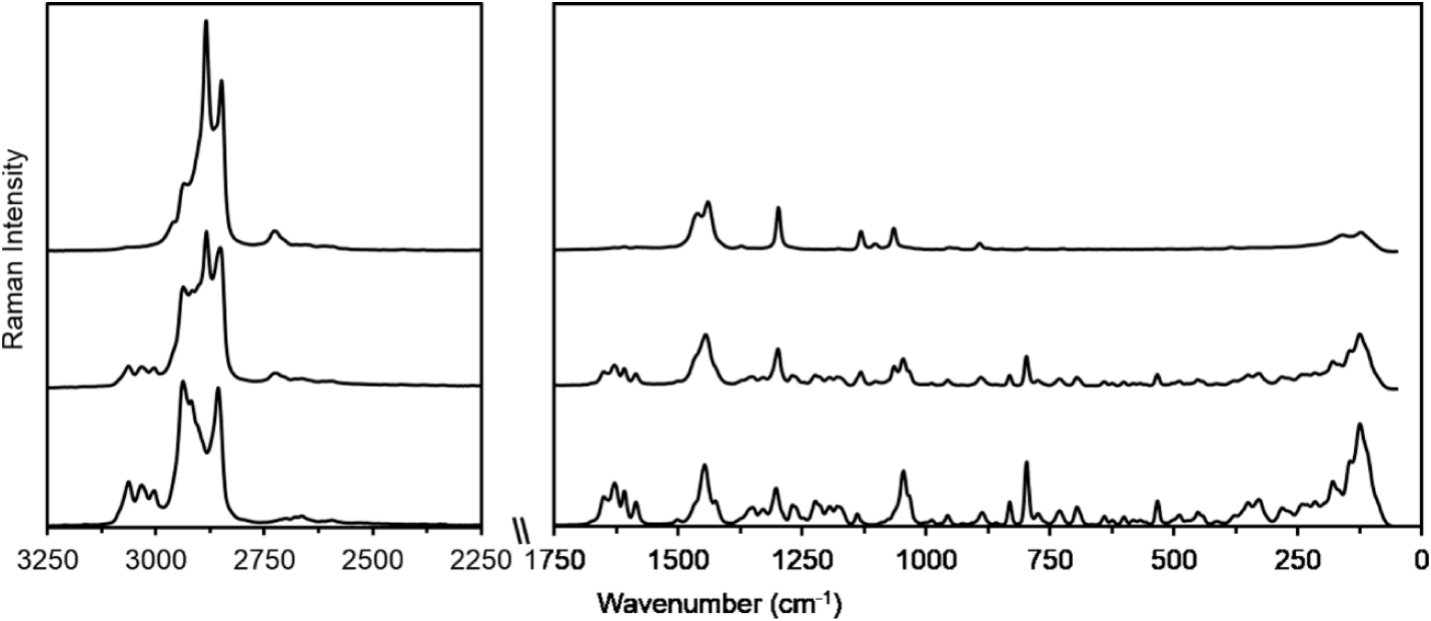
Raman spectra of the PZQ samples. From bottom to top: samples A, B, and C.

#### 3.2.4 Thermogravimetric Analysis (TGA)

Thermogravimetric curves (Figure 10) were collected for samples A, B, and C. A sharp mass loss above 200 °C was found in all the cases, at a temperature above the melting point of PZQ, 136–142 °C (USP, 2019). This thermal event is characteristic of degradation. However, the degradation temperature ranges of the samples were different. For samples A and B, the range was 220–380 °C, as reported in the literature for the degradation of PZQ,^27^ whereas for sample C, the range was shifted to higher temperatures (320–420 °C), suggesting greater thermal stability. These results are consistent with the onset degradation temperatures summarized in Table 3, in which samples B and C presented higher *T*_onset_ values (335 and 337 °C, respectively) compared to sample A (232 °C).

**Figure 10:**
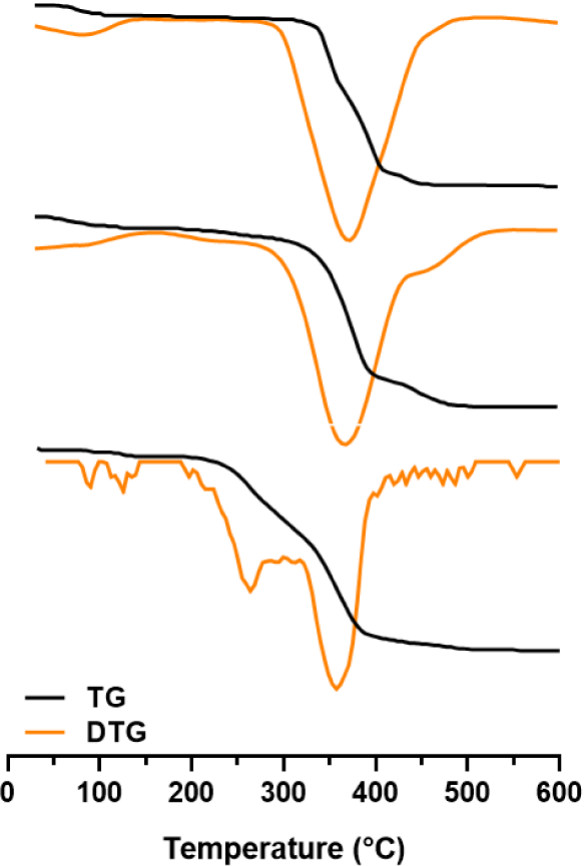
Thermogravimetric analysis (TGA, black curves) and derivative thermogravimetry (DTG, orange curves) profiles of the praziquantel samples. From bottom to top: samples A, B, and C.

**Table 3:** Thermogravimetric Parameters of Praziquantel Samples: Onset Temperature (*T*_onset_) and Mass Loss between 25–100 °C

Sample	*T*_onset_	Mass Loss at 25–100 °C
Sample A	232	1.35 ± 2.00
Sample B	335	7.00% ± 0.96
Sample C	337	6.39% ± 0.88

Another interesting feature observed in the TGA/DTG profiles was the mass loss occurring before the main degradation step. In the 25–100 °C range, samples B and C exhibited comparable mass losses of 7.00% ± 0.96 and 6.39% ± 0.88, respectively (Table 3), which were accompanied by single DTG signals, indicating a unique thermal event associated with the release of absorbed water. In contrast, sample A showed two distinct thermal events prior to degradation: the first one between 25 and 100 °C, with a mass loss of 1.35% ± 2.00 (Table 3), and the second one between 100 and 180 °C, with a mass loss of 2.00% ± 0.14. The presence of two DTG peaks in this region reinforces that more than one volatile component may be released. Together with the lower *T*_onset_ observed for this sample (Table 3), these events suggest the presence of residual solvent and/or absorbed water, which could act as a catalyst for the subsequent degradation of PZQ.

#### 3.2.5 SEM Photomicrographs of the Samples

With respect to particle size and crystal habit, samples A–C did not show great differences (Figure 11). A different particle population is present in the middle of the photomicrograph of sample B. However, this result cannot confirm if these particles are of a different compound.

**Figure 11:**
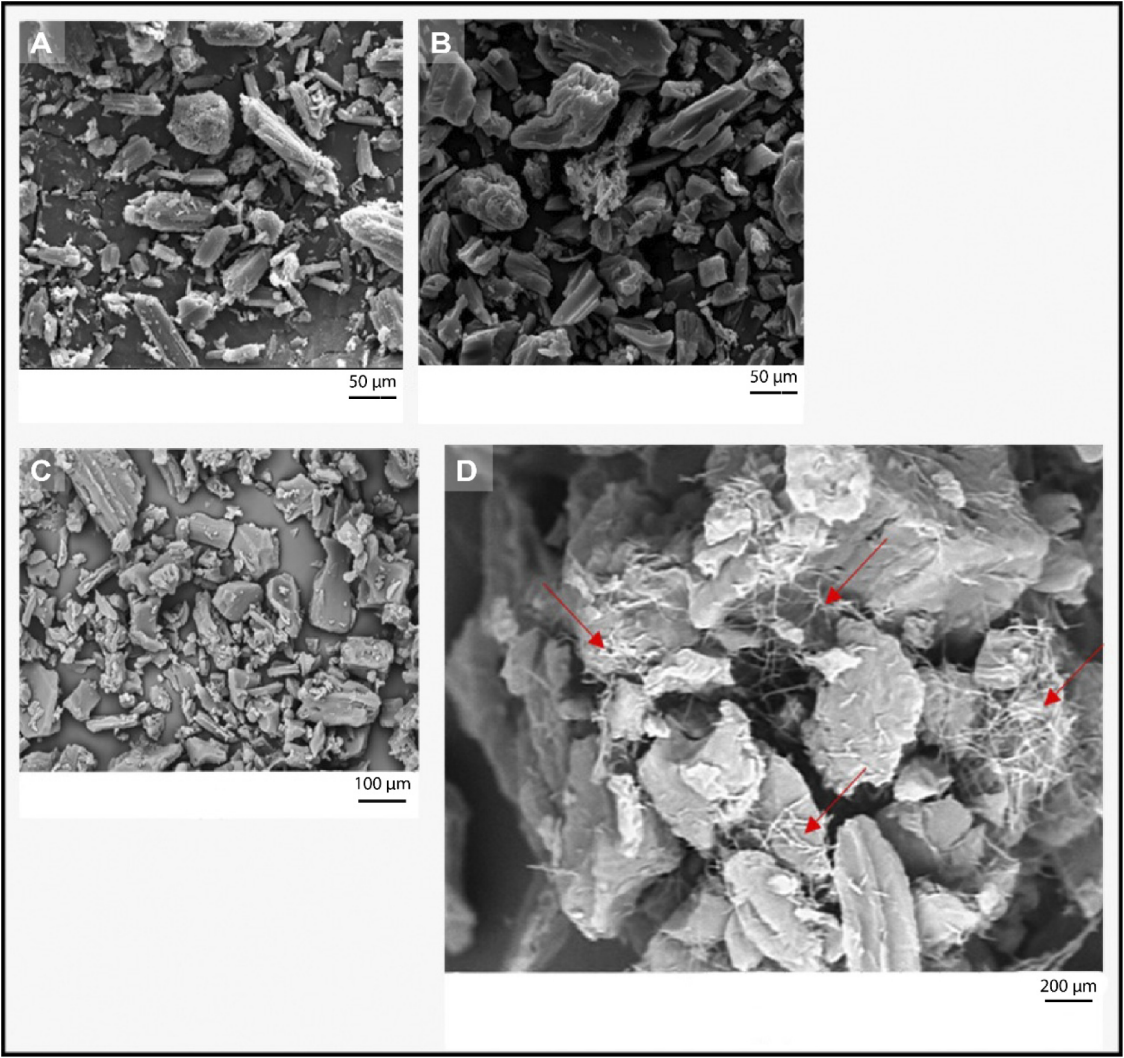
SEM photomicrographs of samples (A) A with 2000× magnification, (B) B with 2000× magnification, (C) C with 1000× magnification, and (D) C with 5000× magnification.

Sample C, with 1000× magnification (Figure 11C) also presented a similar particle shape. However, using a 5000× magnification, it was possible to see very thin needle particles, suggesting crystallization of part of the sample that was prepared with ethanol. Crystallization was also observed on the tablet surface after intrinsic dissolution (dissolution section). It is important to highlight that sample C, after separation with ethanol, presented a light pink color.

#### 3.2.6 NMR Analysis

NMR is a useful technique to identify impurity signals in the samples and has been used to characterize degradation products of PZQ.^28^ NMR spectra were recorded for samples A, B, and C. Interestingly, PZQ was detected as the major compound only in sample A (Figure 12); besides, PZQ was found as a minor compound in sample B and was completely absent in sample C. In both samples A and B, due to the slow rotation of the amide bond in solution, the presence was detected as a mixture of rotamers. This result is in accordance with previous literature reports.^29^

**Figure 12:**
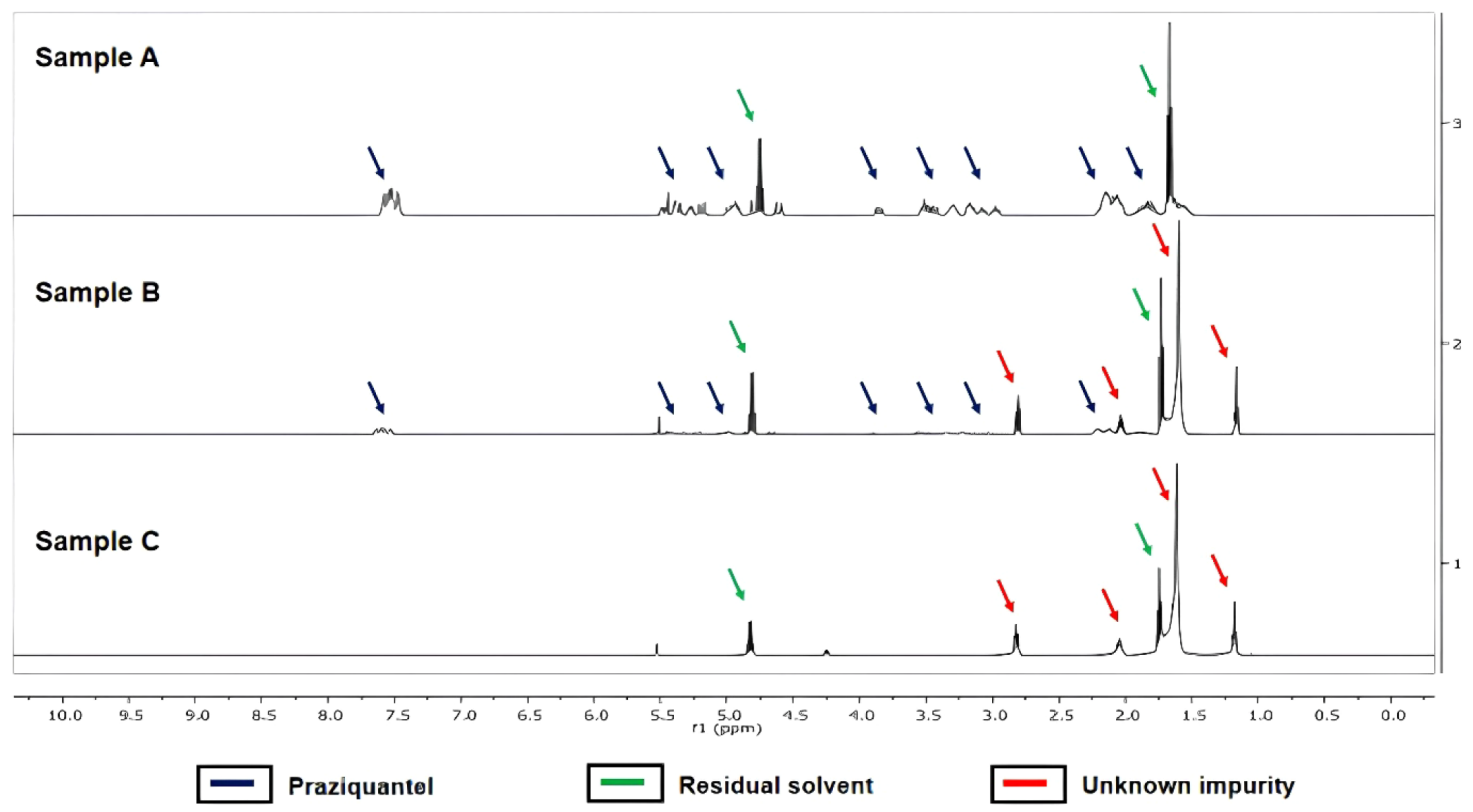
^1^H NMR data of samples A, B, and C.

Sample A also presented another important impurity in its composition, with signals in 7.75 ppm (quartet, *J* = 7.2 Hz, 2H) and in 1.67 ppm (triplet, *J* = 7.2 Hz, 3H). This suggests the contamination of the PZQ sample with ethanol or diethyl ether during its synthesis or even from the crystallization process. Since there are no other contaminants in this sample, we believe that this residual solvent might be responsible for favoring the degradation process, as observed in the TGA analysis.

Moreover, in pink sample B, in addition to PZQ and this solvent, another impurity was detected. This compound presented signals in 2.75 ppm (triplet, *J* = 7.6 Hz, 2H), 2.04 (quintet, *J* = 7.2 Hz, 2H), 1.54 ppm (broad, 24H), and 1.17 (triplet, *J* = 7.1 Hz, 3H). Most interestingly, in sample C, in which the impurity was isolated, only those signals and the solvent contaminant were detected. These signals are characteristic of long aliphatic chains, suggesting a complex degradation process, that might involve photoinitiated radical formation. Moreover, the most deshielded signal (2.75 ppm) suggests the absence of hydrogenated carbon atoms bonded directly to heteroatoms. Interestingly, the impurity did not present any aromatic hydrogen signals, unlike the impurities and the degradation products of PZQ reported in the literature.^28,30^

The ^13^C NMR analysis (Supporting Information) also corroborated these data and revealed other important information regarding the impurity. First, the presence of several carbons in 36.0 ppm, 34.0 ppm, between 31.7 and 31.0 ppm (10 carbons), 24.5 and 18.8 ppm indicated the presence of a long aliphatic chain. Moreover, the presence of a carbonyl in 186.1 ppm suggests the presence of a carboxylic acid. On the other hand, the fact that the impurity is insoluble in deuterium oxide (D_2_O) suggests that this group is not found as a sodium or potassium salt.

### 3.3 Color Change in Sample B During Dissolution

To understand why sample B when in contact with the dissolution medium changed from pink to white, the sample tablet prepared for intrinsic dissolution was evaluated before and after the test. And for a more thorough evaluation, additional tests were done with different media. Figure 13 shows the results.

**Figure 13:**
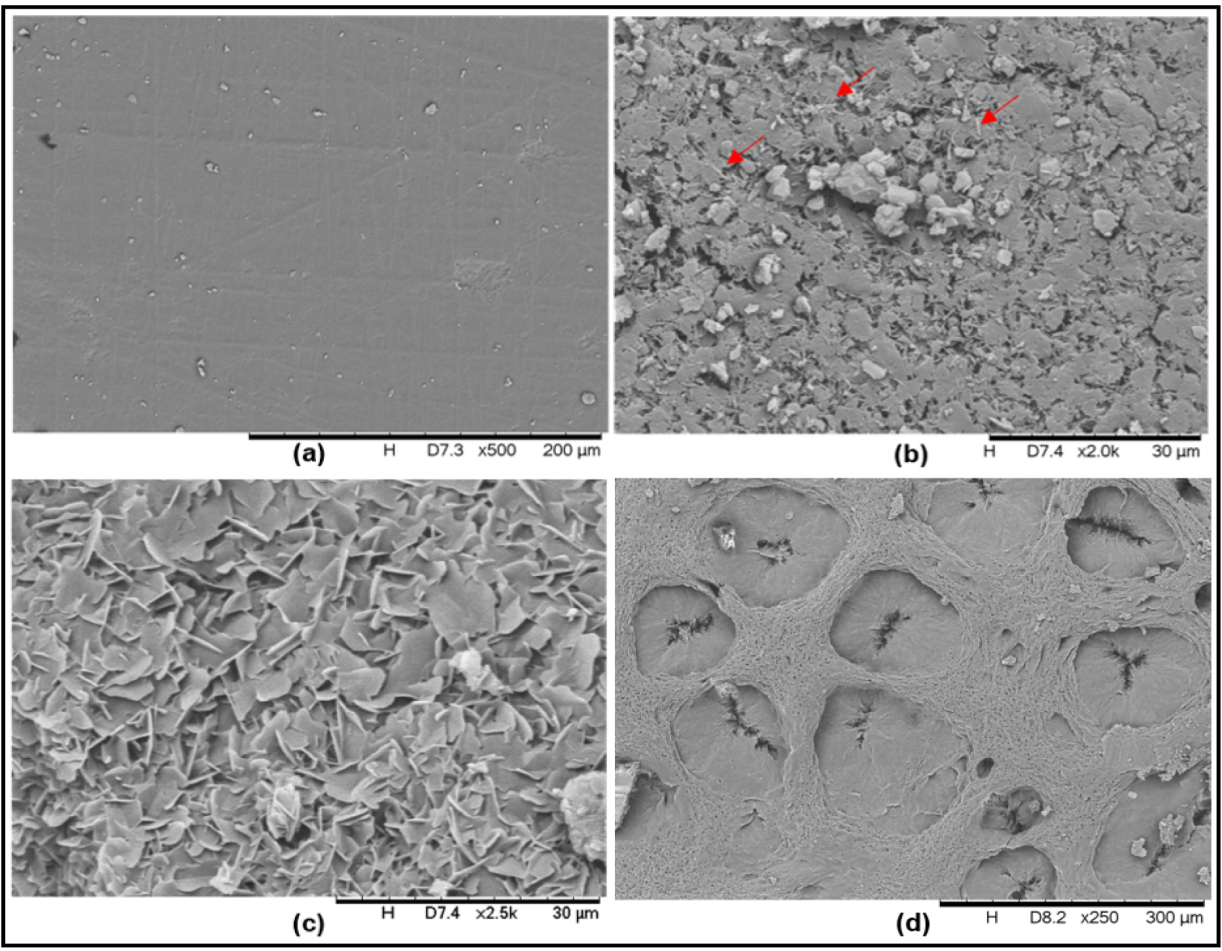
SEM photomicrograph of sample B tablets used for intrinsic dissolution (a) dry, before the test, (b) after the test with water, (c) after the test with HCl 0.1 N pH 1.2, and (d) after the test with HCl 0.1 N pH 1.2 with SLS 0.1% (w/v).

First, good pellet integrity was observed prior to the dissolution test. After the test in water, the formation of small fine particles on the surface was observed, indicating the crystallization of the material. This fact was already observed in Figure 11D. Crystallization becomes more evident after the dissolution test in HCl at pH 1.2. Figure 13d shows the tablet after dissolution in HCl with SLS 0.1% (w/v). The holes that were formed were due to white PZQ that had higher dissolution in the medium. With the addition of SLS, greater dissolution occurred, and it was not possible to see well-formed crystal; they were already partially dissolved.

To confirm that the change in sample color is the result of crystallization of the PZQ degradation product, sample D was evaluated by NMR (Figure 14). PZQ was completely absent in sample D, and this sample showed the same signs as sample C (which contains the degradation product with less crystallinity). In addition, because sample D was obtained after treatment of sample C with HCl and both presented the same NMR pattern, the carboxylic acid group is probably present in the impurity even before the acidic treatment.

**Figure 14:**
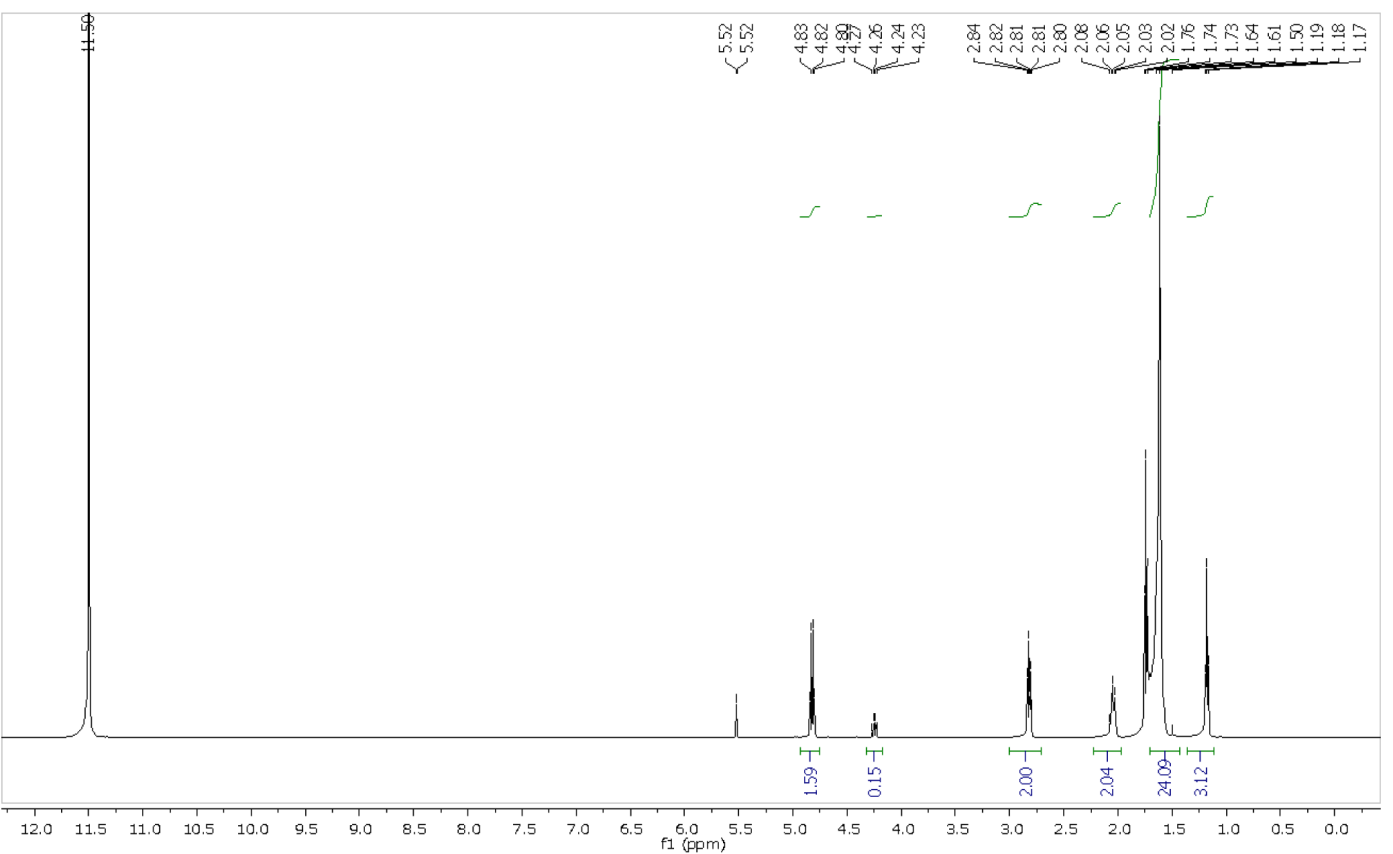
^1^H NMR data of sample D.

## 4 Conclusion

Two batches of PZQ raw material, with different colors (white—sample A and light pink—sample B), were used for the study. After the dissolution tests, in which solubility and wettability were previously evaluated, two other samples were prepared: sample C was isolated from sample B, and sample D was prepared from sample C after exposure to an acid medium. To investigate this phenomenon, several analytical techniques were employed. X-ray powder diffraction showed the presence of a different phase in sample B. With the combination of Raman, infrared, and nuclear magnetic resonance, we proved that the pink color is related to an impurity that is not described in the literature. This impurity presents low crystallinity, and after the contact with water or acid medium, this impurity crystallizes, and the pink color returns to white. So we believe that the pink color of the impurity is related to its microstructure with low crystallinity. Also, we showed that depending on the HPLC method, especially the diluent, this impurity cannot be detected, which in turn is critical for quality control. This paper provided more insight regarding the PZQ color change related to chemical degradation and crystallinity. This fact is critical in the pharmaceutical development of the product and should be investigated during formulation steps and quality control.

## Supplementary Material



## Notes

Catalyst preparation, catalyst characterization, laboratory plant, diffusion coefficients calculation procedure, elemental composition of the catalyst’s samples, characteristic diffraction bands of the clinoptilolite, textural parameters of the catalyst’s samples phase (DOCX)
