# Supplementary material for: First Step into Praziquantel Raw Material Color Change Investigation: The Role of Thermal, Spectroscopic, and Microscopic Techniques
Source: ACS Omega. 2025 Oct 6;10(40):47176–88. doi: 10.1021/acsomega.5c05829 (PMC12529163; doi:10.1021/acsomega.5c05829)
Supplement: Supplementary file 1 [file ao5c05829_si_001.docx]

**First step into praziquantel raw material color change investigation: the role of thermal, spectroscopic and microscopic techniques.**

Livia Deris Prado^a, b^, Silvia Lucia Cuffini^c^, Pedro Pôssa de Castro^d^, Lara Melo Campos^a*^, Giovanni Wilson Amarante^d^, Luiz Fernando Cappa de Oliveira^e^, Helvécio Vinícius Antunes Rocha^a^

^a^ Laboratory of Micro and Nanotechnology, Center of Technological Development in Health, Oswaldo Cruz Foundation, Rio de Janeiro, Brazil;

^b^ Laboratory of Analytical Development and Validation, Farmanguinhos, Oswaldo Cruz Foundation, Rio de Janeiro, Brazil;

^c^Post-graduate Program in Engineering and Science of Materials, Federal University of São Paulo, São José dos Campos, Brazil;

^d^Research Group on Synthetic Methodologies, Department of Chemistry, Federal University of Juiz de Fora, Juiz de Fora, Brazil;

^e^Chemistry Department, Federal University of Juiz de Fora, Juiz de Fora, Brazil

**Supporting Information**

**Table of contents**

[**1.** **NMR data of samples A-D** S3](#_Toc19701500)

# **NMR data of samples A-D**

Figure S1. ^1^H NMR of sample A (500 MHz, TFA-*d*)

Figure S2. ^13^C NMR of sample A (125 MHz, TFA-*d*)

Figure S3. DEPT135 NMR of sample A (125 MHz, TFA-*d*)

Figure S4. ^1^H-^1^H COSY of sample A (TFA-*d*)

Figure S5. ^1^H-^13^C HSQC of sample A (TFA-*d*)

Figure S6. ^1^H-^13^C HMBC of sample A (TFA-*d*)

Figure S7. ^1^H NMR of sample B (500 MHz, TFA-*d*)

Figure S8. ^13^C NMR of sample B (125 MHz, TFA-*d*)

Figure S9. DEPT135 NMR of sample B (125 MHz, TFA-*d*)

Figure S10. ^1^H-^1^H COSY of sample B (TFA-*d*)

Figure S11. ^1^H-^13^C HSQC of sample B (TFA-*d*)

Figure S12. ^1^H-^13^C HMBC of sample B (TFA-*d*)

Figure S13. ^1^H NMR of sample C (500 MHz, TFA-*d*)

Figure S14. ^13^C NMR of sample C (125 MHz, TFA-*d*)

Figure S15. DEPT135 NMR of sample C (125 MHz, TFA-*d*)

Figure S16. ^1^H NMR of sample D (500 MHz, TFA-*d*)

Figure S17. ^13^C NMR of sample D (125 MHz, TFA-*d*)

Figure S18. DEPT135 NMR of sample D (125 MHz, TFA-*d*)
